# Steps toward preregistration of research on research integrity

**DOI:** 10.1186/s41073-021-00108-4

**Published:** 2021-03-01

**Authors:** Klaas Sijtsma, Wilco H. M. Emons, Nicholas H. Steneck, Lex M. Bouter

**Affiliations:** 1 Department of Methodology and Statistics, TSB, Tilburg University, PO Box 90153, 5000 LE Tilburg, The Netherlands; 2 Cito, P.O. Box 1034, 6801 MG Arnhem, The Netherlands; 3 Professor Emeritus of History, University of Michigan, 127 Grandview Drive, Ann Arbor, MI 48103 USA; 4 Department of Philosophy, Faculty of Humanities, Vrije Universiteit Amsterdam, De Boelelaan 1105, 1081 HV Amsterdam, The Netherlands; 5 Department of Epidemiology and Data Science, Amsterdam UMC, Vrije Universiteit Amsterdam, De Boelelaan 1089a, 1081 HV Amsterdam, The Netherlands

**Keywords:** Open Science framework, Paper and poster presenters, Preregistration, Registry for research on the responsible conduct of research, Responsible conduct of research, Study protocol, World conference on research integrity

## Abstract

**Background:**

A proposal to encourage the preregistration of research on research integrity was developed and adopted as the Amsterdam Agenda at the 5th World Conference on Research Integrity (Amsterdam, 2017). This paper reports on the degree to which abstracts of the 6th World Conference in Research Integrity (Hong Kong, 2019) reported on preregistered research.

**Methods:**

Conference registration data on participants presenting a paper or a poster at 6th WCRI were made available to the research team. Because the data set was too small for inferential statistics this report is limited to a basic description of results and some recommendations that should be considered when taking further steps to improve preregistration.

**Results:**

19% of the 308 presenters preregistered their research. Of the 56 usable cases, less than half provided information on the six key elements of the Amsterdam Agenda. Others provided information that invalidated their data, such as an uninformative URL. There was no discernable difference between qualitative and quantitative research.

**Conclusions:**

Some presenters at the WCRI have preregistered their research on research integrity, but further steps are needed to increase frequency and completeness of preregistration. One approach to increase preregistration would be to make it a requirement for research presented at the World Conferences on Research Integrity.

## Background

The purpose of this study was to assess the degree to which paper and poster presenters at the 6th World Conference on Research Integrity ([17]; http://wcri2019.org/) preregistered their research on research integrity using the six key elements described the Amsterdam Agenda (AA; https://www.wcrif.org/guidance/amsterdam-agenda). The AA was developed and adopted at the 5th WCRI ([16]; https://wcrif.org/wcri2017) and recommended for use in research on research integrity. NS and LB were involved in drafting and finalizing the AA. KS analysed the compliance with the AA of the authors of papers and posters accepted for the 6th WCRI (www.wcrif.org/images/2019/PDF/Abstract_book.pdf) and presented his findings during the final plenary session of the conference.

Preregistration can vary from a minimum set of details on the study at issue to a full study protocol, with or without a complete data-analysis plan. For example, Wagenmakers et al. [13] defined preregistration as posting a study protocol including research questions, research design, statistical methods and data-analysis plan in a repository prior to collecting and analyzing the data [13]. WCRI participants could use a number of repositories for preregistering research on research integrity, including: Open Science Framework (OSF) (https://osf.io), Dataverse (https://dataverse.org/), Figshare (https://figshare.com/), and Mendeley (https://www.mendeley.com/). The AA provided guidance on how to register a study with OSF but left the choice of the repository open. We note that the Amsterdam Agenda not only called for preregistering research on research integrity, but also suggested to do so in the Registry for Research on the Responsible Conduct of Research. No one followed that second recommendation and in hindsight, it seems not to be a good idea to have a separate registry for one specific type of study. Furthermore, it is not so clear which studies would belong in such a registry. What is considered as research on research integrity is somewhat a moving target, although probably many would agree that its description by the WCRF Foundation (www.wcrif.org/foundation/mission) accurately characterizes the current situation.

Repositories time-stamp the original document and it later updates so that it can be seen whether potentially data-driven changes of research questions, research design, statistical methods and data-analysis plan were made. The access to the preregistered study protocol can be made public or access can be restricted or embargoed. Based on the relevant literature, our own conceptual analysis, and the interaction we had with the participants before and during the 5th WCRI, we identified the following five core objectives of preregistration.

1. Limiting Researcher Degrees of Freedom. Preregistration limits undesirable flexibility in the analysis and interpretation of the results and thus prevents selective reporting [12]. Several authors have convincingly argued that data analysis in the health sciences as well as in the social and behavioral sciences, economics and in other areas easily elicits activities from researchers known as “*p*-hacking” [14], “fishing” [13], “cherry picking” [5], and “HARKing” (i.e., Hypothesizing After the Results are Known [8];). All these phenomena have in common that they can help to obtain spurious positive results by utilizing undesirable flexibility or ‘researcher degrees of freedom’. Ioannidis [7] convincingly explained why deriving research results from analyzing data without a prespecified plan produces very often results that fail to stand firm upon replication (also, [9, 11]).
2. Ensuring Replicability. The detailed study protocol that preregistration requires, ensures that others can repeat the study based on the information the researcher preregistered [10].
3. Revealing Unpublished Studies. Preregistration that is publicly accessible allows authors of systematic reviews to assess the magnitude of publication bias due to (often negative) studies of which the results weren’t published [4].
4. An improved version of preregistration are registered reports (RR [15];). In RR the introduction and method sections are submitted to a journal before data collection starts. Reviewers assess the study plan, including the research questions, research design, statistical methods and data-analysis steps envisaged, and the journal editor makes a decision about acceptance of the research for publication solely on the basis of the relevance of the study and the soundness of its methods. This version of preregistration highlights a fourth core idea:
5. Greater Review Benefit. Reviewers’ comments on RR submissions may improve the study because its design can still be adapted [3].
6. Protecting Reviewers and Editors from Bias. RRs also prevent the journal editor and the reviewers from letting the research outcomes, unknown at the time of review and decision making, affect their decision of whether to publish the study, and thus prevents publication bias and overrepresentation of positive results. This benefit is supported by the fact that final publications preceded by RRs mention negative results substantially more often than comparable articles that were not preregistered [1]. Nosek et al. [10] support preregistering research; that is, committing oneself to an a priori study protocol. They conclude that without preregistration statistical analyses are rather meaningless and could better be avoided.

The World Conferences on Research Integrity foster the exchange of information and discussion about responsible conduct of research (https://wcrif.org/). Beginning with the 2010 Singapore Statement, the World Conferences have adopted conference statements that are designed to foster or improve integrity in research. The Amsterdam Agenda was developed during the 5th conference in 2017, with the goal of establishing a Registry for Research on the Responsible Conduct of Research (RRRCR; https://osf.io/jbqkv/; part of OSF). The registry was focused especially on researchers engaged in studying research integrity in all its facets and aimed explicitly at having researchers use the six key elements outlined in the AA when developing their study protocol. These key elements were made available to the participants of the 6th WCRI and can be found on the organization’s website (https://www.wcrif.org/guidance/amsterdam-agenda); they are:

- Problem. The integrity researcher should describe the shortcomings one addresses, such as selective reporting or the misuse of statistics.
- Impact. One should estimate the impact the target shortcomings have on the trustworthiness of research, how they affect the responsible use of research funds, etc.
- Intervention. Researchers should explain how they plan to address shortcomings when identified. Examples of actions are future quality checks, training of researchers to prevent future shortcomings, and encouragement of responsible behavior.
- Hypothesis or Anticipated Outcomes. Researchers should explain the changes in future research they expect their intervention will cause.
- Assessment. One should explain the planning of hypothesis testing and assessing whether outcomes are as expected.
- Data sharing. Finally, the registry requires researchers to clarify how data, either qualitative or quantitative, will be shared with other researchers.

To preregister a study, researchers were expected to provide information on each of the six key elements, upload a full study protocol, and, once the study was completed, upload a data set from the study and reports describing results. Preregistration of studies on which an abstract was submitted for the 6th WCRI was voluntary.

## Methods

The data used for this study were extracted from participant registration files that the organizers of the 6th WCRI set up for administrative purposes. The files used were dated 24 April 2019, 5 weeks before the start of the Conference, and contained information supplied by the participants when they registered for the Conference. The following independent variables were identified ad hoc for our study:

1. Presentation Mode (paper or poster),
2. Early Career Scholar (no or yes),
3. Topic of the paper or poster (nine topic categories, six of which were available to the participants at the time they submitted their abstract and three were based on coding the entries in the free text field the participant filled out),
4. Category of Research (qualitative or quantitative research, both using empirical data and together called empirical research, or non-empirical research not using empirical data, such as an opinion paper or a review paper, and description of cases; these three categories were available at the time of abstract submission),
5. Continent affiliated with the presenter, and
6. Discipline in which the researcher was active (eight disciplines, defined ad hoc based on the entries in a free text field the participant filled out).

The three dependent variables were:

1. Preregistration: no or yes,
2. The Repository Used: OSF RRRCR, OSF other, or another repository, and
3. Completeness: the degree to which preregistration was complete ranging from no entries to six entries.

Data for 308 accepted paper and poster abstracts were available. The analysis that follows is based on information from participants who indicated that they had preregistered the research on which they reported in the abstract. Participants who preregistered were asked to provide the URL for the repository to enable assessment of the preregistration. Some of the abstracts shared one or more coauthors or concerned different aspects of the same study, possibly introducing dependence between a few cases. The sample size was too small to tackle this problem. Also, the subset of the sample actually preregistering was too small to allow estimation of associations between independent and dependent variables with acceptable precision. Therefore, we only provide descriptive statistics.

## Results

Table 1 (upper panel) shows that 19% (58) of the 308 studies the accepted abstracts were preregistered. Of the 58 studies that were preregistered, 2 did not provide information for the dependent variables Repository Used and Completeness of preregistration, so that 56 studies (lower panel) remained for further analysis with respect to Repository Used and Completeness. For Repository Used, of the 56 studies that provided additional information about the preregistration, 41 reported using OSF RRRCR (21) or OSF other (20). For Completeness of preregistration, 2 of the 56 preregistered studies did not provide information about any of the six key elements from the AA, and 21 of the 56 preregistered studies provided information for at least 1 out of the 6 key elements of the AA; specifically, 7 provided information about 1—4 key elements and 14 provided information about 5—6 key elements. Of these 21 studies, 20 were qualitative or quantitative empirical studies. Based on 207 qualitative (*n* = 82) and quantitative (*n* = 125) empirical studies in total (Table 2), these 20 studies represent 10%. The one non-empirical study that preregistered provided information on 1 AA element (not tabulated).

**Table 1:** Information about Preregistration (Yes/No) for all Presented Studies, and Information about The Repository Used for Preregistration, and Completeness of the Registration for the Preregistered Studies

Registration Characteristic	Number of Studies	Percentage of All Studies	Percentage of Preregistered Studies
Preregistration			
No	250	81%	–
Yes	58	19%	–
Results for preregistered Studies (*n* = 56)^a^			
Repository Used			
OSF RRRCR	21	7%	37%
OSF other	20	6%	36%
Another repository	15	5%	27%
Completeness			
0 key elements	2	1%	4%
1–4 key elemements	7	2%	12%
5–6 key elements	14	4%	25%
No evidence^b^	7	2%	12%
Different title	2	1%	4%
Required sign-in	10	3%	18%
Wrong URL	14	4%	25%

^a^ Two participants out of the 58 in the upper panel provided no information with respect to dependent variables Registry or Completeness of preregistration, hence the number of preregistered studies for which enough information was available was 56

^b^ “No Evidence” means that presenters claimed they preregistered while in fact there was no evidence they did

**Table 2:** Number and Percentage of Preregistered Studies for Different Types of Research (*N* = 308)

Research Type	Number of Presented Studies	Number of Studies Preregistered (% in brackets)	
Qualitative	82	19	(23)
Quantitative	125	33	(26)
Non-Empirical	101	6	(6)

For the other 33 studies that the authors claimed they preregistered the results were as follows. For 7 studies, we found no evidence of preregistration; 2 studies provided information for a project that had a title different from the accepted abstract while it was unclear whether it concerned the same study as the one accepted as abstract; for 10 studies, the repository required signing-in codes we did not have; and 14 studies provided an URL that produced an error message or led to a website irrelevant to useful registry.

Based on Table 2, we conclude that qualitative and quantitative empirical research did not clearly differ with respect to Preregistration—of both research types one quarter preregistered—but non-empirical projects were not often preregistered (6% out of 101 projects did). This is understandable, given that non-empirical studies referred to opinion and review papers, course descriptions, and so on.

Table 3 shows for each discipline the number and the percentage of preregistration, for all studies and for empirical studies only. Researchers in the areas of research ethics and research integrity preregistered remarkably less than researchers from other disciplines. The explanation is that ethics and integrity researchers are more often engaged in non-empirical research (51% out of 76 projects) than researchers from other research areas (27% out of the other 229 projects). It may be noted that researchers from the human sciences and from medicine and health preregistered less than half of their empirical studies. Because we have a small sample, it is too early for drawing definitive conclusions.

**Table 3:** Numbers and Percentages of Preregistered Studies for Different Disciplines (*N* = 305 - three missing values for discipline), Among all Studies and Among the Empirical Studies

Study Characteristic	Number of Presented Papers and Posters		Number of Preregistered Studies	
	Total (*n* = 305)	Empirical (*n* = 204)	Among All Studies (% in brackets)	Among Empirical Studies (% in brackets)
Ethics, Integrity	76	37	6 (8)	6 (16)
Natural Sciences	20	12	4 (20)	2 (17)
Human Sciences	55	50	15 (27)	15 (30)
Library, Information	8	6	1 (13)	1 (17)
Medicine, Health	112	79	27 (24)	24 (30)
Publishing, Journals	16	10	1 (6)	1 (10)
Governance, Funding	15	7	3 (20)	2 (29)
Non-Academic	3	3	1 (33)	1 (33)

## Discussion

Preregistration options are most developed for experimental research (e.g www.clinicaltrials.gov and https://osf.io/) and preregistration of non-empirical studies is difficult and probably not very useful. In addition, systematic reviews (www.crd.york.ac.uk/prospero/) and qualitative research [6] preregistration options recently became available and one could always upload a study protocol before start of the data-collection to any repository that time stamps. Because preregistration is still under development, we decided not to exclude particular studies from preregistering for the 6th WCRI.

Only 58 (19%) of the studies claimed to be preregistered, but for 33 of these we were unable to verify this. The low percentage of preregistration is probably attributable to a number of factors: 1) Preregistration was recommended, not required. Since preregistration takes time, some may have decided that the benefits of preregistration were not worth the extra effort. 2) Some studies may have already started their data collection when the abstract was submitted. 3) Preregistration is at odds with a long tradition of accepting flexibility and data-driven data analyses as adequate research practices. Preregistration involves a different working routine. Changing common practices takes time. 4) Finally, it is possible that the six key elements outlined in the AA are still too abstract or not suitable to all fields of research. The six elements may need further clarification and a more concrete operationalization with a view to make preregistration easier.

The plan to study the degree to which 6th WCRI paper and poster presenters preregister so that we would be able to monitor preregistration frequency in future conferences originated shortly before the conference. The planning of our study close to the 6th WCRI and its ad hoc character meant that the data had already been collected. Because of time pressure, we missed the opportunity to preregister our study or its data-analysis plan.

Based on this initial analysis of the success in implementing the recommendations of the AA, a follow-up study is planned among accepted abstracts for 7th WCRI. This study will be preregistered and we will define the variables for analysis and research hypotheses a priori. Potential participants wishing to present a paper or a poster can then provide the relevant information for the research directly when registering for the conference. To obtain results that are more valid, we will also try to profit from best practices derived from other similar work (e.g. [2]). We intend to give a boost to preregistration in RRRCR for the 7thWCRI 2022 in Cape Town, South Africa, and present the results of the next research round during the conference and in print. For the 7^th^ we will strongly recommend preregistration of both quantitative and qualitative empirical studies but for the 8th WCRI we’ll consider to make it mandatory [6, 10].

## Data Availability

The datasets used and/or analyzed during the current study are available from the corresponding author on reasonable request.

## Abbreviations

AA: Amsterdam Agenda
OSF: Open science framework
RR: Registered reports
RRI: Research on research integrity
RRRCR: Registry for Research on the Responsible Conduct of Research
WCRI: World Conference on Research Integrity
